# Esophageal submucosal hematoma developed after endovascular surgery for unruptured cerebral aneurysm under general anesthesia: a case report

**DOI:** 10.1186/s40981-017-0124-3

**Published:** 2017-10-03

**Authors:** Sachiko Ito, Shihoko Iwata, Izumi Kondo, Motoyo Iwade, Makoto Ozaki, Tatsuya Ishikawa, Takakazu Kawamata

**Affiliations:** 1 Department of Anesthesiology, Tokyo Women’s Medical University, Tokyo, 162-8666 Japan; 2 Department of Neurosurgery, Tokyo Women’s Medical University, Tokyo, 162-8666 Japan

**Keywords:** Esophageal submucosal hematoma, Coil embolization, Cerebral aneurysm, Antiplatelet and anticoagulant therapies, Cough during tracheal extubation

## Abstract

**Background:**

Esophageal submucosal hematoma is a rare complication after endovascular surgery. We report a case of an esophageal submucosal hematoma which may have been caused by rigorous cough during extubation.

**Case presentation:**

A 75-year-old woman underwent endovascular treatment for unruptured cerebral aneurysm under general anesthesia. The patient received aspirin and clopidogrel before surgery and heparin during surgery. Activated clotting time was 316 s at the end of surgery. Protamine was not administered and continuous infusion of argatroban was started after surgery. She had a rigorous cough during removal of the tracheal tube and reported retrosternal discomfort postoperatively. She developed hemorrhagic shock after massive hematemesis. A diagnosis of esophageal submucosal hematoma was made by endoscopic examination and computed tomography. Hemostasis was achieved by compression with a Sengstaken-Blakemore tube and endoscopic cauterization. Blood pressure was recovered by blood transfusion. Endoscopic examination performed 7 days after surgery showed that esophageal submucosal hematoma had almost disappeared and slough had adhered to the mucosal laceration. The patient showed good recovery and was discharged 21 days after surgery.

**Conclusions:**

Careful extubation and postoperative observation are required in patients receiving antiplatelet and anticoagulant therapy.

## Background

Esophageal submucosal hematoma is a rare condition [1]. Previous reports showed that it may develop after endovascular surgery, which requires postoperative anticoagulation therapy. Chest pain is the most frequent complaint [2], which often misleads a diagnosis such as ischemic heart disease and aortic dissection. We report a case who had hematemesis and developed hemorrhagic shock after endovascular surgery for unruptured cerebral aneurysm under general anesthesia. Prompt endoscopic examination enabled early diagnosis and successful treatment. We discuss the causes of and potential preventive strategies for esophageal submucosal hematoma as well as important reminders at the time of onset and diagnosis and postoperative management. We obtained written informed consent from the patient to publish this case report.

## Case presentation

A 75-year-old woman (154 cm, 49 kg) underwent coil embolization of an unruptured cerebral aneurysm under general anesthesia. Her medical history and preoperative complications were unremarkable. Preoperative laboratory data were within normal limits, with no coagulation abnormalities. Aspirin 200 mg and clopidogrel 300 mg, and aspirin 100 mg and clopidogrel 75 mg were administered orally on the day before, and in the morning of the day of surgery, respectively.

General anesthesia was induced with target-controlled infusion of propofol and remifentanil 0.5 μg/kg/min. After infusion of rocuronium 50 mg, tracheal intubation was performed uneventfully, followed by smooth insertion of a nasogastric tube, which was left open to room air until the completion of surgery, and there was no spontaneous reflux of gastric contents. Anesthesia was maintained with target-controlled infusion of propofol and remifentanil 0.1 μg/kg/min with an inhalational oxygen concentration of 37% under standard monitoring as well as direct radial artery pressure monitoring. Blood pressure and heart rate were stable with continuous infusion of phenylephrine 0.4–0.5 mg/h.

Heparin 400 units were intravenously administered at the beginning of surgery, followed by 500 units during surgery. Activated clotting time (ACT) was 141, 238, 275, and 316 s before and 5 and 40 min after starting surgery, at the end of surgery. Protamine was not administered. Continuous infusion of argatroban 0.8 μg/kg/min was started on completion of surgery.

Fentanyl 100 μg was administered 10 min before surgery completion, and continuous infusion of propofol, remifentanil, and phenylephrine was discontinued at the completion of surgery. The patient started to wake 5 min after the completion of surgery, and her systolic blood pressure increased to 160 mmHg and pulse rate to 90 bpm. The nasogastric tube was removed without applying negative pressure after aspirating a small amount of gastric fluid not containing apparent blood components. Sputum was seen in the tracheal tube, which was suctioned. Following this, the patient had a stronger cough. The tracheal tube was removed 12 min after the completion of surgery after confirming recovery of consciousness. After extubation, there was no nausea, vomiting, or strong cough. The patient was stable with a blood pressure of 140/60 mmHg and pulse rate of around 90 bpm, and returned to the ICU 20 min after extubation. Total operative time was 75 min, and total anesthesia time was 130 min.

**Fig. 1:**
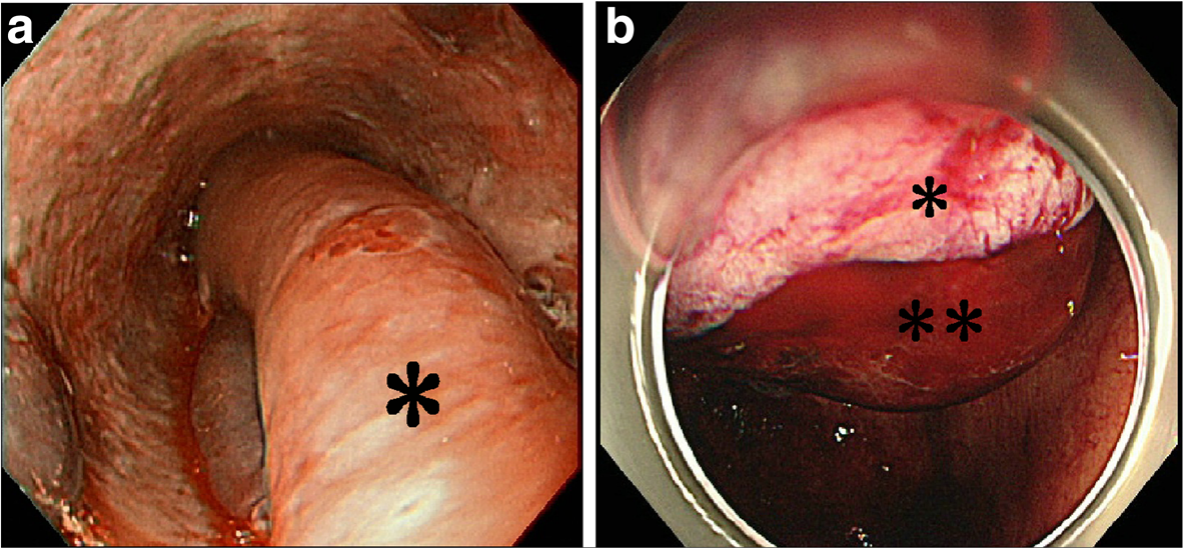
Upper gastrointestinal endoscopy images immediately after hematemesis. **a** A longitudinal extension of reddish or wine-colored mucosal thickening (asterisk), obstructing the esophagus, is seen from the midesophagus to the esophagogastric junction. **b** The submucosal hematoma (two asterisks) is displacing the esophageal mucosa (asterisk) toward the gastric cavity at the esophagogastric junction. A part of the displaced mucosa has a laceration with bleeding

**Fig. 2:**
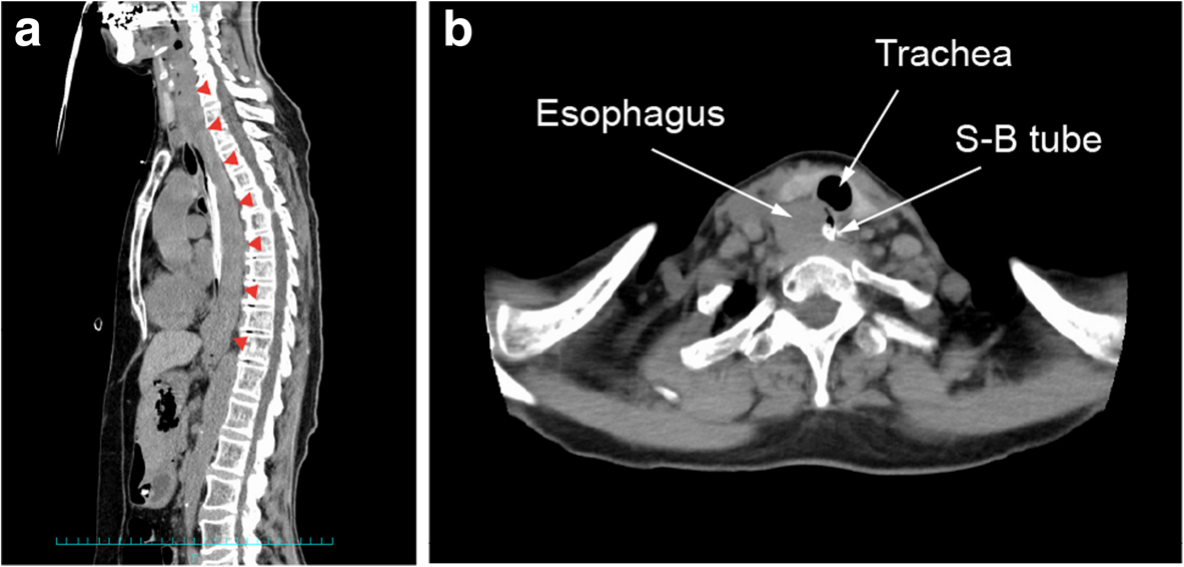
Cervical and thoracic CT images after insertion of Sengstaken-Blakemore tube. **a** Sagittal section: the soft tissue shadow is filled and dilatation can be seen in the posterior wall over the entire length of the esophagus (red arrows), ventrally displacing the lumen. **b** Cross-section: the cervical esophagus is dilated, displacing the trachea toward the left side

The patient started to complain of retrosternal discomfort 24 min after return to the ICU. At that time, her blood pressure was 140/60 mmHg and pulse rate was around 90 bpm. The patient started to complain of nausea 48 min after returning to the ICU. Her systolic blood pressure decreased to 70 mmHg 51 min after returning to the ICU, and the patient had a hematemesis of approximately 200 g of fresh blood. And, the ACT was 280 s. An upper gastrointestinal endoscope was inserted immediately to identify the bleeding point and achieve hemostasis, which showed submucosal thickening extending from the midesophagus to the esophagogastric junction, which was due to the submucosal hematoma (Fig. 1a: endoscopic image). At the esophagogastric junction, the esophageal mucosa was being displaced into the gastric cavity by the submucosal hematoma (Fig. 1b: endoscopic image). There was a bleeding laceration in part of the mucosa, for which pressure hemostasis was performed with a Sengstaken-Blakemore tube. Continuous infusion of argatroban was discontinued and protamine was administered, and the ACT decreased to 126 s. During the endoscopic examination, the submucosal hematoma was extending toward the oral side, and it became a hematoma extending from the esophagogastric junction to the oral cavity at the end of the endoscopic procedure. The hematemesis continued, and the amount of bleeding reached 900 g, and the level of hemoglobin decreased to 7.5 g/dL. A plain computed tomography (CT) was performed to assess the extent of the hematoma, which showed dilatation of the entire esophagus and the soft tissue shadow filled on the dorsal side that was ventrally displacing the lumen (Fig. 2a: CT sagittal section). These findings, along with the endoscopy results, were consistent with the diagnosis of esophageal submucosal hematoma. An isodense area suggesting hematoma was also observed in the gastric cavity. In addition, the dilated cervical esophagus was displacing the trachea (Fig. 2b: CT cross-section). Considering that the patient would have a greater risk of aspirating blood into the trachea if the hematoma ruptured, therefore, we performed tracheal intubation. Observation during the tracheal intubation showed edema at the arytenoid region. After compression with the Sengstaken-Blakemore tube for 1 h, hemostasis was achieved by cauterizing some parts of the mucosal laceration at the esophagogastric junction using the upper gastrointestinal endoscope. Up to this point, the patient was given fluid resuscitation for the decreased blood pressure due to the hemorrhage and was transfused with 10 units of red blood cells and 2 units of fresh frozen plasma.

**Fig. 3:**
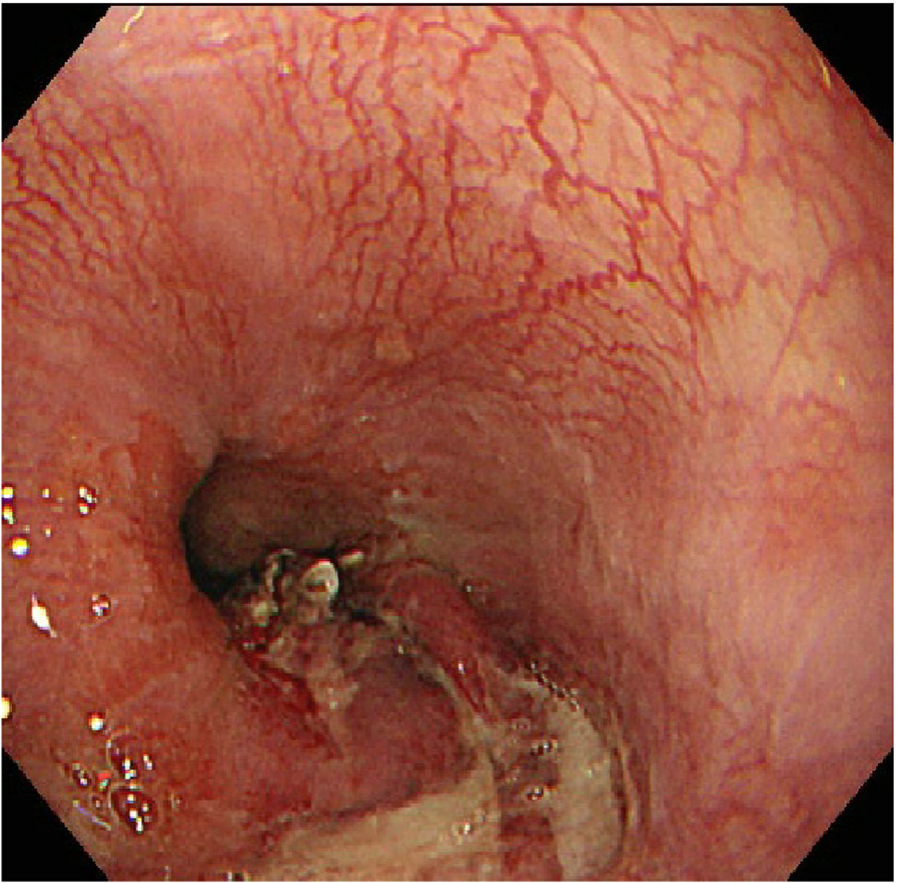
Upper gastrointestinal endoscopy image 7 days after surgery. The esophageal submucosal had almost disappeared, and the slough had adhered to the mucosal laceration at the esophagogastric junction

The patient was fasted and started on omeprazole 40 mg/day; antiplatelet therapy was discontinued. Upper gastrointestinal endoscopy performed on the day after surgery confirmed the absence of expansion of the hematoma or recurrent bleeding. Neck and thoracic plain CT performed 4 days after surgery showed reduced dilatation of the esophagus and improvement in displacement of the trachea, and thus the tracheal tube was removed 5 days after surgery. Upper gastrointestinal endoscopy performed 7 days after surgery showed that the esophageal submucosal hematoma had almost disappeared and the slough had adhered to the mucosal laceration at the esophagogastric junction (Fig. 3). Oral intake was resumed on the same day. The patient showed good recovery and was discharged 21 days after surgery. The patient did not develop thrombotic complications until discharge, although she did not receive antiplatelet or anticoagulant therapy.

Esophageal submucosal hematoma is a rare condition in the spectrum of esophageal injury including Mallory-Weiss syndrome and Boerhaave syndrome [3]. These conditions are similar in that they are induced by a sudden increase in esophageal pressure. While Mallory-Weiss syndrome and Boerhaave syndrome are usually associated with vomiting, patients with esophageal submucosal hematoma do not always present with vomiting [3, 4]. Classification of the causes of esophageal submucosal hematoma into idiopathic (spontaneous) and traumatic has originally reported by Freeman AH [2]. The idiopathic causes of esophageal submucosal hematoma include a sudden increase in the esophageal pressure not only by vomiting or vomiting reflex but also by coughing and sneezing. Sometimes, the causes are unknown [3–5]. The traumatic causes of esophageal submucosal hematoma include direct or blunt injury caused by medical apparatus inserted into the esophagus [6], insertion of gastric tube [7], and accidental swallowing of foreign objects. Most reported cases with esophageal submucosal hematoma were of patients receiving antiplatelet drugs or who had abnormal coagulation [6–8]. Therefore, bleeding tendency is a contributing factor to the aggravation of esophageal submucosal hematoma [5].

In the present case, the patient had a stronger cough during the period from the time when the patient woke up from general anesthesia to the time of extubation of the tracheal tube, especially when we suctioned sputum from the tracheal tube. We considered that an increased abdominal pressure due to the cough led to elevated esophageal pressure that could be a cause of the esophageal submucosal dissection or esophageal mucosal injury. During the upper gastrointestinal endoscopic examination performed immediately after hematemesis, the submucosal hematoma was observed to be extending toward the upper esophagus with repeated hematemesis, suggesting that the elevated esophageal pressure was the primary cause of the esophageal submucosal hematoma and its expansion in the present case. Although cough is a rare cause of esophageal submucosal hematoma, Cao et al. reported a case of esophageal submucosal hematoma occurring after persistent coughing for 1 week in a patient with viral bronchitis [5].

The nasogastric tube was inserted into the stomach after introducing general anesthesia and left open to room air. Direct damage to the mucosa by the nasogastric tube was considered unlikely, because the tube had been inserted into the stomach without resistance and that bloody secretion had not been aspirated from the tube before extubation. Fujimoto et al. reported a case of esophageal submucosal hematoma possibly caused by gastric tube removal with sustained negative pressure [7]. In the present case, aspiration was performed only once before extubation. However, if the tube had been placed around the esophagogastric junction, mucosal injury due to aspiration might have caused the submucosal hematoma.

In general, when performing coil embolization for an unruptured cerebral aneurysm, antiplatelet therapy is started preoperatively and anticoagulant therapy is started intraoperatively to prevent thrombotic complications [9, 10], However, as there are no established guidelines for preventive treatment, the methods vary between institutions and according to morphology of aneurysms. In our department of neurosurgery, we use two antiplatelet drugs in combination, we administer heparin during surgery to maintain an ACT of 200 s or longer, and we perform continuous infusion of argatroban for 48 h after surgery as anticoagulant therapy. We compared the use of anticoagulant therapy in the present case with that in two previously reported cases of esophageal submucosal hematoma after coil embolization for unruptured cerebral aneurysm [7]. Regarding antiplatelet drugs, one patient used aspirin [7], while the other used aspirin and clopidogrel in the same combination [1] as was used in the present case. Regarding anticoagulant therapy, the initial dose of heparin and ACT during surgery were similar in the three cases; however, the ACT at the completion of surgery was longer in the present case (316 s) than in the other two cases (239 and 265 s, respectively), and the continuous infusion of argatroban was performed only in the present case. Therefore, relatively intensive antithrombotic therapy performed in the present case was considered to have an influence on the occurrence of extensive submucosal hematoma of the esophagus and subsequent massive hematemesis with hemorrhagic shock.

Hematemesis, as a symptom of esophageal submucosal hematoma, results from rupture of the most vulnerable part of the mucosa due to expansion of the hematoma [6]. The bleeding point in the present case was the lower part of the esophagus. Anatomically, the mucosa of the lower part of the esophagus is prone to injury under the influence of intrathoracic or esophageal pressure [5]. In the present case, it was considered that the mucosa of the lower part of the esophagus became fragile and ruptured due to an increased pressure by coughing at the time of extubation. In esophageal submucosal hematoma, the amount of hematemesis is usually small. Shim et al. reported that, among 119 patients with esophageal submucosal hematoma reported since 1968, 11 had more than 500 g hematemesis with hypotension or bleeding that required transfusion of at least 4 units [4].

The present study suggests that coughing at the time of extubation can be a cause of esophageal submucosal hematoma or mucosal rupture. Therefore, clinicians should be very careful about the risk of esophageal submucosal hematoma, especially when treating patients receiving antiplatelet or anticoagulant therapy under general anesthesia. We retrospectively considered that we could have reduced cough if we had suctioned the sputum in the tracheal tube under deep anesthesia. In addition, the use of nasogastric tube should be carefully considered, because its insertion and removal is associated with risk.

## Conclusions

The prognosis of esophageal submucosal hematoma is good. However, patients receiving antiplatelet or anticoagulant therapy have a risk of massive bleeding that requires urgent treatment. Clinicians should be aware of the risk of esophageal submucosal hematoma in postoperative management of patients undergoing coil embolization for cerebral aneurysm.

## Abbreviations

ACT: Activated clotting time
CT: Computed tomography
